# The Fundamentals of the NP-Gram Method for the Characterisation of Pyrolysis Oils Based on the Estimated Boiling Points of Pyrolysis Products from Polypropylene

**DOI:** 10.3390/polym17131855

**Published:** 2025-07-02

**Authors:** Mihai Brebu, Katsuhide Murata

**Affiliations:** 1 “Petru Poni” Institute of Macromolecular Chemistry, 41A Grigore Ghica Voda Alley, 700487 Iași, Romania; 2 Toyohashi Chamber of Commerce and Industry, Toyohashi 440-8508, Japan; muratakh@yahoo.co.jp

**Keywords:** hydrocarbon boiling points, pyrolysis products, polypropylene, NP-gram

## Abstract

The pyrolysis of polymers is a thermal processing method largely used to convert polymeric waste into valuable products such as oils and carbonaceous residues. The NP-gram method (NP standing for normal paraffins) is useful for the global characterisation of pyrolysis oils with complex composition. Here, we present the fundamental of this method, which is based on the concept of “carbon number”, in conjunction with the boiling point and the chromatographic retention time of chemical compounds. Polypropylene was selected as the model polymer due to its simple mechanism of thermal degradation. The boiling points of the main compounds in polypropylene pyrolysis oil were estimated based on the equations of Egloff and Wiener. A good correspondence was obtained for the estimated boiling points and the position of the compounds in the gas chromatogram. A distinction was made between the number of carbon atoms in the molecule and the corresponding carbon number used in characterisation of pyrolysis oils by NP-gram. Correlation with the chromatographic retention index was also discussed. The application of the NP-gram method for different polymers was also presented.

## 1. Introduction

The boiling point is one of the most important thermal properties of compounds since it can be used to calculate many other thermo-chemical properties. Interest in correlating the boiling points of hydrocarbons with the molecular structure in homologue series was reported as early as 1842, with the aim of calculating values for compounds lacking in experimental data. It was in 1939 that Egloff collated and evaluated all previous attempts in finding equations to estimate the boiling points of hydrocarbons [1] and in 1940 that he proposed his own equations [2]. Egloff and co-workers found a variation in boiling point (T) with the number of carbon atoms in hydrocarbons (nC) according to Equation (1), where *a*, *b* and *k* are empirical constants. They also found that *a* and *b* remain constant for aliphatic hydrocarbons while only *k* changes from series to series, and they reported that Equation (2) can predict to within ~1 °C the boiling points of normal paraffins (Tn), expressed in degrees Kelvin.

T = a log(nC + b) + k(1)

T*n* = 745.42 log(nC + 4.4) − 416.31 (2)

A few years after Egloff’s reports, Wiener became interested in the correlation of physical properties of hydrocarbons with structural arrangement of atoms in molecules, and in 1947 he reported his Equation (3) on calculating the shift in properties for a group of isomers compared to the corresponding normal paraffins [3]. He found that various physical properties such as the heat of isomerization and heat of vaporisation [4], boiling point at different pressures [5] or aniline point, surface tension and specific dispersion [6] follow Equation (3), where p and ω are structural variables while *k* and *b* are empirical constants. For the shift in boiling point of branched paraffins compared to normal ones, they reported Equation (4).

ΔG = G*n* − G*iso* = *k* Δω/nC2 + *b* Δp (3)

ΔT = T*n* − T*iso* = 98Δω/nC2 + 5.5 Δp (4)

The polarity number, p, was defined by Wiener as “the number of pairs of carbon atoms which are separated by three carbon-carbon bonds” and was interpreted as “a semi-quantitative measure of intramolecular attraction forces transmitted through the carbon chain”. The path number, ω, was considered a measure of the compactness of a molecule; that is, a smaller ω means a higher compactness of the molecule. Weiner defined ω as the sum of the distances between any two carbon atoms in the molecule, in terms of carbon–carbon bonds, and ω can be calculated by multiplying the number of carbon atoms on one side of any bond by those on the other side and summing these values for all bonds. Wiener gave the example below (5) to show the calculation of p and ω for 2,3-dimethylpentane. Normal paraffins p and ω were found to follow the recurrence Formula (6).

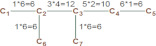
pairs 3 bonds apart: C_1_C_4_, C_1_C_7_, C_2_C_5_, C_7_C_5_, C_6_C_4_, C_6_C_7_; p=6 ω = 6+12+10+6+6+6 = 46(5)

p*_n_*_C_ = nC − 3; ω*_n_*_C_ = (nC − 1)nC(nC + 1)/6 (6)

Wiener’s structural variables p and ω became a strong point of interest for mathematicians, and they are generally known as Wiener polarity index and Wiener index, respectively. The Wiener index is now one of the most frequently used molecular-shape descriptors in graph theory, together with the Hosoya (Z) index [7] and Randić (molecular connectivity) index [8]. Physical–chemical interpretations of the Wiener index [9,10] as well as of the Wiener polarity index [11] have been thoroughly studied. Li extended the Wiener indices in attempt to improve their usefulness in QSAR/QSPR (quantitative structure–activity/structure–property relationships) analysis [12,13]. Abrams and Lauderdale further explored the Wiener index for embedded graphs [14]. Liu and Lee used Wiener’s parameters for predictions on thermal pressure coefficients for normal and branched paraffins based on their molecular structure [15].

Yu et al. successfully correlated the normal boiling points, critical constants and refractive index of acyclic halogen derivatives, ethers and amines, based on a modified Wiener equation; however, this approach gave poor results for alcohols [16]. Chan et al. developed an empirical method to determine the boiling point for a diverse range of organic molecules based on effective molecular surface area, determined using a generalised Egloff approach [17]. Gamba et al. developed correlations to predict critical parameters of n-alkanes and isoalkanes based on normal boiling point [18]. Satou et al. used group contributions to estimate the boiling point of hydrocarbons based on high-performance liquid chromatography (HPLC) and gas chromatography (GC) [19]. Santak and Conduit used an artificial neural network to train and then predict the boiling point of isoalkanes; however, this was limited to up to 13 carbon atoms [20]. Qu et al. used neural networks to fit within 6K the literature data on the boiling points of organic compounds [21].

Zhang et al. used the molecular inductive effect index to correlate the boiling point of the paraffins with the length of the molecular chain [22]. Cao and Cao proposed the intermolecular interaction index as a topological parameter for the intermolecular dispersion forces affecting the boiling point of alkanes [23]. They also reported on general equations for linear and non-linear variation in properties with the number of carbon atoms in the molecule [24]. Here, we present our estimation of boiling points for the main compounds from the thermal degradation of polypropylene and the implications in GC characterisation of pyrolysis oils by the NP-gram method.

## 2. Materials and Methods

Amounts of 10 g of polystyrene, polypropylene and polyethylene were pyrolysed in a semibatch reactor, heating the furnace by 10 °C/min up to 360, 380 and 420 °C, respectively, and then maintaining it isothermally until a condensable product was observed.

The liquid pyrolysis products were analysed by gas chromatography coupled with mass spectrometry (GC-MSD), injecting ~0.2 μL oils in the inlet port (heated at 250 °C) of an 6890 N-5975 inert XL equipment from Agilent, Santa Clara, CA, USA. The separation was performed under 1 mL/min He flow using a standard HP5-MS (30 m × 0.25 mm × 0.25 μm) column which was heated by 10 °C/min from 40 °C (after an isothermal step for 2 min) up to 320 °C. The qualitative identification of the compounds was performed based on the NIST20 database, considering a minimum quality of recognition of 85%.

## 3. Degradation Products of Polypropylene

**Scheme 1:**
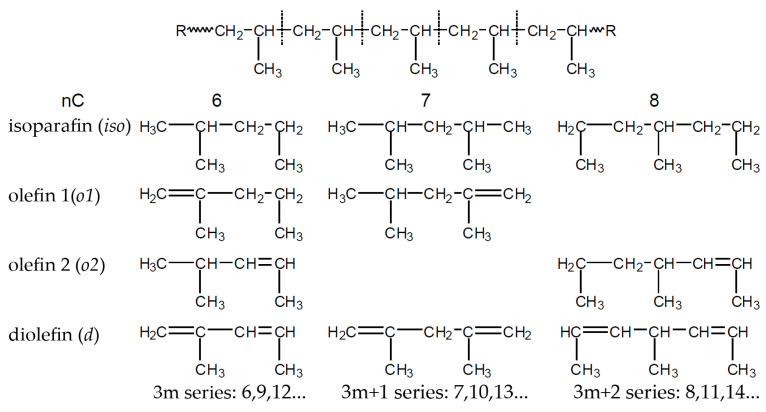
Structures of the main types of compounds produced from thermal degradation of PP.

The thermal degradation of polypropylene (PP) preferentially occurs by scissions of the macromolecular backbone at the positions marked in Scheme 1. One scission is needed to release the chain end units, while two scissions are necessary to liberate both ends of a small random fragment along the main chain. When chain-end scission or random scissions at the preferred positions occur, compounds of 3m type are produced. When one of the two random scissions is not at the preferred position, compounds of 3m+1 or 3m+2 type are produced. Scissions of the C-C bond in the main chain lead to the formation of a saturated and an unsaturated end. Depending on the placement of the unsaturation relative to the broken bond, three types of compounds are formed: saturated paraffins (iso) and mono-unsaturated (*o1* and *o2*) or double-unsaturated (d) olefins. The compounds from the 3m+1 series will have only *o1*-type olefins, while the compounds from the 3m+2 series will only be of *o2*-type olefin. Scheme 1 shows the structure of the compounds resulting from the degradation of PP at the most favourable positions.

The polarity number, p, and the path number, ω, for the isoparaffins in the PP degradation compounds were determined according to Wiener’s rules (5) based on the structures in Scheme 1, extended up to nC27. The obtained values are shown in Table 1, together with the values for the equivalent normal paraffins that were calculated according to Wiener Equation (6). Then, the shift in boiling points from normal to isoparaffins was calculated with Wiener Equation (4).

It was found that p and ω follow regular behaviour with the number of carbon atoms in the homologue series of PP degradation compounds. Therefore, the recurrence formula could be determined to calculate p and ω for each PP series of interest, as described by Equation (7).

3m series: p = (4nC − 15)/3; ω = (nC3 + 3nC2 − 6nC)/9 3m+1 series: p = (4nC − 16)/3; ω = (nC3 + 3nC2 − 9nC + 5)/9 3m+2 series: p = (4nC − 14)/3; ω = (nC3 + 3nC2 − 3nC − 5)/9(7)

The boiling points of isoparaffin degradation products of PP were calculated by applying the temperature shifts calculated based on the Wiener method (last column in Table 1) to the boiling point of corresponding normal paraffins, calculated based on Egloff Equation (2)—second column in Table 2. Correction factors were used when going from saturated to unsaturated hydrocarbons, depending on their structure, according to Equation (8). These factors were considered based on Egloff’s relations on 2-methyl-1-alkenes (for *o1* isomers), 2-alkenes (for *o2* isomers) and on summations of 2-methyl-1-alkene end with 2-alkene end (for *d* isomers of 3m series), two 2-methyl-1-alkene ends (for *d* isomers of 3m+1 series) and two 2-alkene ends (for *d* isomers of 3m+2 series).

3m series: *o1*: + 1.7 °C; *o2*: −0.3 °C; *d*: + 1.4 °C 3m+1 series: *o1*: + 1.7 °C; *d*: + 3.4 °C 3m+2 series: *o2*: −0.3 °C; *d*: −0.6 °C (8)

In the calculation of the compactness of molecules, the Wiener index ω considers that all distances between the carbon atoms in the molecule are the same. However, this does not apply to unsaturated hydrocarbons, since the bond length varies according to the hybridization of the carbon atoms. Li defined a new valence Wiener index that considers the relative size of bonds in the molecule in an attempt to improve correlations for unsaturated hydrocarbons; however, despite the relatively good regression coefficients, the standard errors were not very good [25,26]. Therefore, in our work, we preferred to use the Egloff deviations from the saturated paraffins, knowing that this also has limitations since only the effect of the terminal groups (where the double bonds are placed in PP degradation products) is considered. Results are given in Table 2 and compared to the experimental values reported by the NIST database [27].

The boiling points of normal paraffins calculated by the Egloff equation are in good agreement with the experimental values, as reported by Egloff up to nC18. With new NIST data on heavier hydrocarbons, we can say that the boiling points start to be overestimated above nC19 (italics in Table 2). However, the validity of the Egloff equation could be satisfactory when extended from nC19 up to nC22 (1 < ΔT < 2 °C, green in Table 2), and to a lesser extent (ΔT > 3 °C, red in Table 2) above nC25. While the calculated boiling points of normal paraffins can be compared with present databases, experimental information is lacking for isoparaffins, olefins and diolefins; hence, the theoretical calculation is very important.

**Figure 1:**
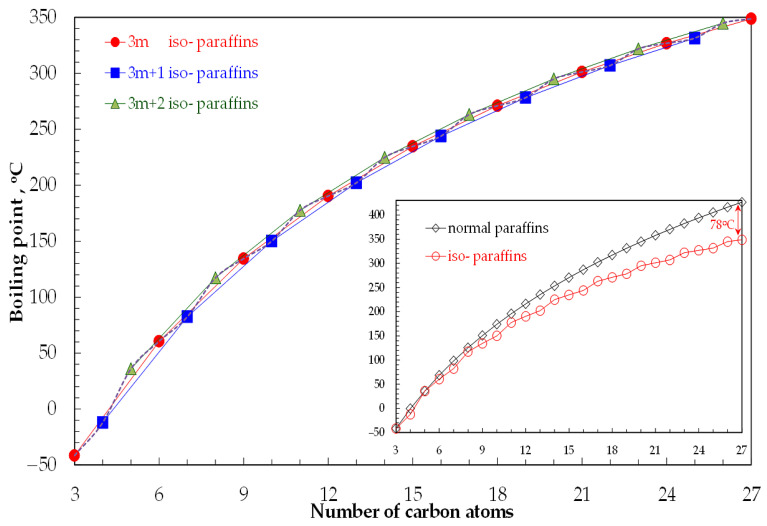
The variation in estimated boiling point of the normal and isoparaffin products of PP with the number of carbon atoms in the molecule.

The boiling points of isoparaffins significantly decrease compared to those of the corresponding normal paraffins above nC9, so a 77.7 °C temperature drop was estimated for nC27 (P9 nonamer)—Figure 1. The isoparaffins followed a zig-zag pattern when considering the entire range of PP degradation products. However, smooth, regular patterns were found for each individual series, with the 3m lying between 3m+2 and 3m+1 ones. This could be explained by the fact that the short chain ends (four methyl groups) of the compounds in the 3m+1 series increase the compactness of the molecules and hence decrease the boiling points. The compounds in the 3m+2 series are also symmetric but have two longer chain ends (two propyl groups in addition to two methyl ones) with higher flexibility. The increased van der Waals interactions between these larger molecules require more energy to vaporise them, and thus the boiling temperature is higher. The propylene oligomers in the 3m series have three methyl and one propyl groups at the chain ends, and thus they lie in between.

**Table 2:** The calculated and NIST-reported boiling points for normal paraffins (*n*) and for iso (*iso*), olefin 1 (*o1*), olefin 2 (*o2*) and diolefin (*d*) degradation products of PP; in bold – PP oligomers.

nC	T*_n_*_calc_	T*_n_*_NIST_	T*_iso_*_calc_	T*_iso_*_NIST_	T*_o1_*_calc_ ^a^	T*_o1_*_NIST_	T*_o2_*_calc_	T*_o2_*_NIST_	T*_d_*_calc_	T*_d_*_NIST_	Cn ^b^	Ri ^c^
2	−88.5	−88.5									2	200–299
3	−41.5	−42.0	−41.5 (Tn)		**−48.0 P1**	−47.5					3	300–399
4	−0.5	−0.1	−12.1	−11.1	−10.4	−6.4					4	400–499
5	35.9	36.1	35.9 (Tn)	27.9			35.6	36.5			5	500–599
6	68.7	68.8	60.5	60.9	**62.2 P2**	61.8	60.2	58.4	61.9	75.9	6	600–699
7	98.4	98.4	82.4	80.6	84.1	81.5			85.8	89.6	7	700–799
8	125.6	125.6	117.3	117.8			117.0	113.5	116.7	117.4	8	800–899
9	150.7	150.8	134.4	132.9	**136.1 P3**	134.0	134.1		135.8		9	900–999
10	174.0	174.1	150.1		151.8				153.5		10	1000–1099
11	195.7	195.5	177.6				177.3		177.0			
12	216.1	216.4	190.4		**192.1 P4**		190.1		191.8		11	1100–1199
13	235.3	234.5	202.2		203.9				205.6		12	1200–1299
14	253.4	252.9	224.9				224.6		224.3		13	1300–1399
15	270.5	270.6	234.7		**236.4 P5**		234.4		236.1			
16	286.8	286.6	243.9		245.6				247.3		14	1400–1499
17	302.3	301.9	263.2				262.9		262.6		15	1500–1599
18	317.0	316.2	271.0		**272.7 P6**		270.7		272.4			
*19*	*331.2*	*329.8*	278.3		280.0				281.7		16	1600–1699
*20*	*344.7*	*343.1*	295.1				294.8		294.5		17	1700–1799
*21*	*357.7*	*356.6*	301.2		**302.9 P7**		300.9		302.6			
*22*	*370.2*	*368.7*	307.0		308.7				310.4		18	1800–1899
*23*	*382.3*	*380.1*	321.9				321.6		321.3			
*24*	*393.9*	*391.4*	326.8		**328.5 P8**		326.5		328.2		19	1900–1999
*25*	*405.1*	*402.0*	331.5		333.2				334.8			
*26*	*415.9*		344.7						344.2		20	2000–2999
*27*	*426.4*		348.7		**350.4 P9**		348.8		350.1		21	2100–2199

^a^ P1–P8: propylene monomer—octamer; ^b^ Cn: carbon number of compounds, from GC chromatograms in Figure 2; ^c^ Ri: Kovats retention index range.

Table 2 shows that most PP degradation products have lower boiling points compared to their corresponding normal paraffins, and the difference significantly increases with the number of carbon atoms of the molecule in the series. This is mainly due to the presence of many side methyl groups on the chain that affect the compactness of the molecule. Indeed, Table 1 shows that while the path number, ω, is strongly affected by the number of the bonds in the chain (ω is about 800 times larger for nC27 compared to nC3), its increase is much smaller for isoparaffins. Hence, the more compact isomers in PP products will boil at much lower temperatures due to the lower frequency of molecular collisions. The polarity number, p, has much lower variation and thus a lesser effect on the variation in boiling points with increasing size of the molecule. Unsaturation also did not significantly affect the boiling points, as could be seen from the low correction factors in Equation (8).

A consequence of this phenomenon is that propylene oligomers boil in the temperature range of much lower normal paraffins, that is, P2 (nC6, 62.2 °C) in the range of nC5–nC6 (36.1–68.7 °C), P3 (nC9, 136.2 °C) in the range of nC8–nC9 (125.7–150.8 °C), P4 (nC12, 192.1 °C) in the range of nC10–nC11 (174.1–195.5 °C), P5 (nC15, 236.4 °C) at nC13–nC14 (234.5–252.9 °C), P6 (nC18, 272.7 °C) at nC15–nC16 (270.6–286.6 °C) and P7 (nC21, 302.9 °C) at nC17–nC18 (301.9–316.2 °C).

This behaviour requires the introduction of a parameter named carbon number (Cn), which represents the number of carbon atoms in the highest normal paraffin that boils below the molecule of interest. That is, all hydrocarbons with a boiling point between the nC and (n + 1)C normal paraffins have the carbon number Cn. Since in gas chromatography the non-polar compounds leave non-polar columns in order of their increasing boiling point, the relative chromatographic position of hydrocarbons is constant irrespective of analysis parameters, as represented by the Kovats retention index [28]. From this point of view, the carbon number of a compound is its Kováts retention index rounded down to an integer and divided by a factor of 100. For example, 2-methyl-1-pentene, the polypropylene P2 dimer, with a boiling point of 61.8 °C, has a Kovats retention index of 580, corresponding to its C5 carbon number. One can see from the last column in Table 2 that the Cn carbon numbers of propylene oligomers are as follows: P1: Cn2; P2: Cn5; P3: Cn8; P4: Cn10; P5: Cn13; P6: Cn15; P7: Cn17; P8: Cn18.

**Figure 2:**
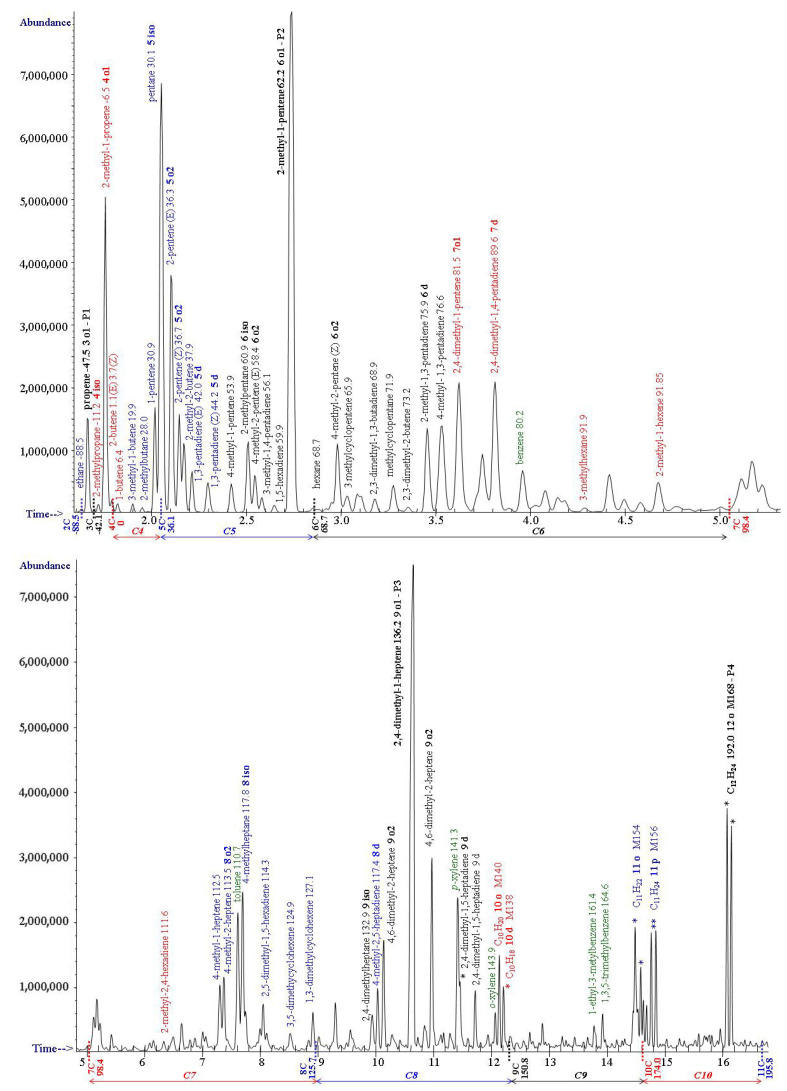

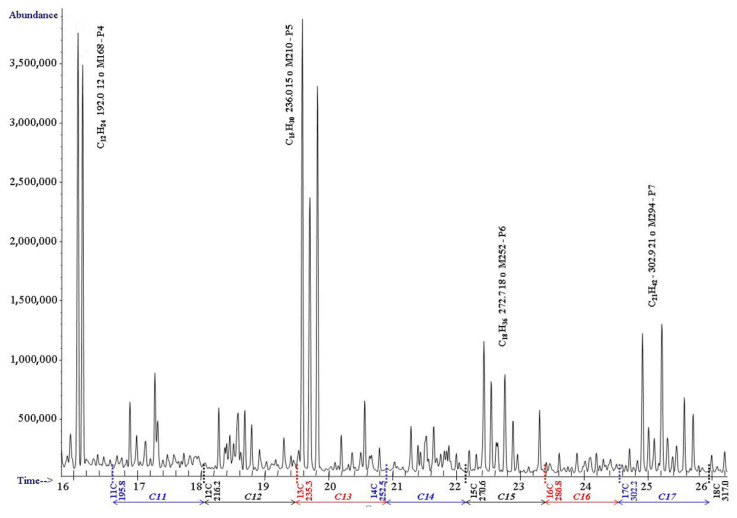
GC-MSD chromatogram of a PP degradation oil and the position of normal paraffins (from PE degradation oil) used as external reference compounds (black, red, blue colours for matching the compounds with the carbon number; green for aromatic compounds).

While the Kovats retention index is largely used for the qualitative identification of compounds according to retention index database, the introduction of carbon number (Cn) is of great importance for the global characterisation of complex mixtures of volatile chemical compounds. For this, Murata and Makino [29] proposed the NP-gram method (NP stands for normal paraffin), which represents the integrative chromatographic peak area of the compounds versus their carbon number. NP-grams are based on gas chromatographic analysis and consider that all compounds appearing in the chromatogram in the retention time range between two successive normal paraffins belong to the carbon number of the lower normal paraffin. This is represented in Figure 2 by the arrows and the carbon indices below the retention time axis. The sum of all peak percentage area in a retention time range is represented versus the corresponding carbon number. There is no need for any qualitative identification of compounds in the chromatogram in order to obtain the NP-gram; moreover, the method allows NP-grams of different pyrolysis oils to be plotted and compared on the same figure. If the chromatogram is obtained using a flame ionisation detector (FID), the peak area approximates the amount of compounds satisfactorily. In this case, the NP-gram shows the mass percentage distribution of compounds in the mixture (pyrolysis oil), which is equivalent to the simulated distillation curves. The NP-gram method has the advantages of very small amounts of sample required, easy change in chromatographic analysis parameters for the optimum separation of compounds and a short analysis time. When a mass selective (MS) detector is used for the identification of GC peaks, the percentage peak area is not equivalent to the percentage mass composition; therefore, the NP-gram does not show the true mass distribution of compounds. However, it does allow relative comparisons among similar samples.

The NP-grams offer the rapid visualisation of the distribution of the compounds according to the carbon numbers, which is equivalent to the boiling points. In addition, the NP-grams can replace the distillation curves, which are usually considered for the characterisation of petrochemical products, offering the advantage of rapid analysis of only very small amounts of samples. Details will be discussed further based on the chromatographic characterisation of PP degradation oils.

Figure 2 shows a detailed chromatogram of a PP pyrolysis oil with the names and experimental boiling points of the main identified compounds. The boiling points of normal paraffins and their positions in the chromatogram, determined using PE oil as the external reference, are shown on the retention time axes. GC analysis confirms that the PP degradation oil contains mainly compounds of the types shown in Scheme 1, from which the 3m *o1* series (propylene oligomers) are the most important. Some general behaviours of the series of hydrocarbons can be observed in Figure 2. For the same number of carbon atoms, the PP products leave the column in the *p* < *o1* < *o2* < *d* order; however, some *o*- isomers (E) have lower boiling points and leave earlier. For lower numbers of carbon atoms, the aliphatic compounds are distributed on both sides of the corresponding normal paraffins (shown below the time axis), and usually the branched compounds are to the left (lower boiling points) and the unsaturated ones are to the right. However, above nC9, the compounds appeared at a lower carbon number, as was also shown in Table 2.

Since polar compounds such as those from torrefaction and the pyrolysis of biomass interact less with non-polar columns, their retention time, and hence retention index and carbon number in the NP-gram curves, is lower than of the corresponding paraffins with similar boiling points, with the difference increasing with the polarity of the compound—Table 3.

**Figure 3:**
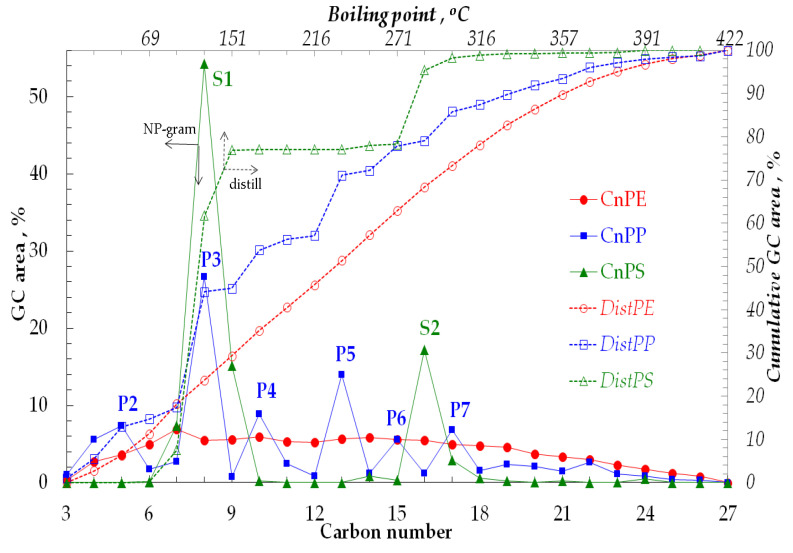
The NP-grams and simulated distillation curves for pyrolysis oils of PE, PP and PS.

Figure 3 shows the NP-grams and the simulated distillation curves for the pyrolysis oils of polyethylene (PE), polypropylene (PP) and polystyrene (PS). It is clear that propylene oligomers are the main compounds in the pyrolysis oil and appear as peaks at positions other than the numbers of their number of carbon atoms, similar to the data in Table 2. This is different to the broad distribution of compounds in PE oil, where random scission is not selective, and to the narrow distribution of styrene monomers and dimers that comes from strongly selective scissions of the macromolecular backbone. Figure 3 also shows that compared with the simulated distillation curves, the NP-grams offer the advantage of rapid visualisation of the quantitative distribution of compounds in the pyrolysis oils.

## 4. Conclusions

The estimation of the boiling points of the main compounds in the pyrolysis oils of polypropylene was made based on the equations proposed by Egloff and Wiener. It was found that the validity of the Egloff equation could be satisfactory extended from nC18 up to nC22, and to lesser extent up to nC25. Recurrence formulae were determined that help in calculating the Wiener structural parameters p and ω for the PP degradation compounds. The gas chromatography–mass selective analysis of PP degradation oil shows that the positions of compounds in the chromatogram are in good agreement with the estimated values of boiling points. The highly branched structure of PP degradation compounds strongly decreases the boiling points and the propylene oligomers have lower carbon numbers than the number of their carbon atoms. The NP-gram method reveals a different distribution of the pyrolysis compounds from polyethylene, polypropylene and polystyrene.

## Figures and Tables

**Table 1:** The polarity number (p) and path number (ω) determined for normal paraffins (*n*) and for isoparaffins (*iso*) found as the main PP degradation products.

nC	p*_n_* nC − 3	ω*_n_* (nC − 1)nC(nC + 1)/6	p*_iso_*	ω*_iso_*	ΔT*n*-*iso* *
3m series					
3	0	4	-	-	0.00
6	3	35	3	32	8.17
9	6	120	7	102	16.28
12	9	286	11	232	25.75
15	12	560	15	440	35.77
18	15	969	19	744	46.06
21	18	1540	23	1162	56.50
24	21	2300	27	1712	67.04
27	24	3276	31	2412	77.65
3m+1 series					
4	1	10	0	9	11.63
7	4	56	4	48	16.00
10	7	165	8	135	23.90
13	10	364	12	288	33.07
16	13	680	16	525	42.84
19	16	1140	20	864	52.93
22	19	1771	24	1323	63.21
25	22	2600	28	1920	73.62
3m+2 series					
5	2	20	-	-	-
8	5	84	6	75	8.28
11	8	220	10	184	18.16
14	11	455	14	365	28.50
17	14	816	18	636	39.04
20	17	1330	22	1015	49.68
23	20	2024	26	1520	60.37
26	23	2925	30	2169	71.10

* ΔT*n*-*iso* = T*n* − T*iso* = 98(ω*_n_* − ω*_iso_*)/nC^2^ + 5.5(p*_n_* − p*_iso_*).

**Table 3:** The boiling points (bp), retention index (RI) and carbon number (nC) of polar compounds from the torrefaction and pyrolysis of biomass, compared with the equivalent normal paraffins [30,31].

Compound	bp	RI	nC	Equivalent Normal Paraffin	bp
Acetic acid	117.9	624	6	Heptane	98.4
Acetol (hydroxyacetone)	145	655	6	Octane	125.7
2,3-butanediol	177	791	7	Decane	174
Furfural	161.7	828	8	Nonane	150.8
Furfuryl alcohol	171	850	8	Nonane	150.8
Phenol	181.7	975	9	Decane	174
Guaiacol	205	1086	10	Undecane	195.8
Syringol	261	1350	13	Tetradecane	252

## Data Availability

Data are contained within the article.
